# Subclinical hypothyroidism and height loss in relation to the status of thyroid cysts: a prospective study

**DOI:** 10.1265/ehpm.25-00458

**Published:** 2026-04-03

**Authors:** Yuji Shimizu, Nagisa Sasaki, Asuka Oyama, Yuko Noguchi, Mutsumi Matsuu-Matsuyama, Koichiro Hamada, Shin-Ya Kawashiri, Hirotomo Yamanashi, Seiko Nakamichi, Yasuhiro Nagata, Takahiro Maeda, Naomi Hayashida

**Affiliations:** 1 Department of General Medicine, Nagasaki University Graduate School of Biomedical Sciences, Nagasaki, Japan; 2 Epidemiology Section, Division of Public Health, Osaka Institute of Public Health, Osaka, Japan; 3 Department of Community Medicine, Nagasaki University Graduate School of Biomedical Sciences, Nagasaki, Japan; 4 Department of Health Society and Statistics, Atomic Bomb Disease Institute, Nagasaki University, Nagasaki, Japan; 5 Leading Medical Research Core Unit, Nagasaki University Graduate School of Biomedical Sciences, Nagasaki, Japan; 6 Nagasaki University Health Center, Nagasaki, Japan

**Keywords:** Endothelial repair, Height loss, Latent damage, Subclinical hypothyroidism, Thyroid cysts, Thyroid hormone, Thyroid stimulating hormone

## Abstract

**Background:**

Despite numerous recent studies linking height loss to the risk of cardiovascular disease, the underlying biological mechanisms are poorly understood. Subclinical hypothyroidism (SCH) has been positively associated with height loss in individuals with low-normal range free thyroxine (FT4) levels. This association may stem from impaired endothelial repair caused by an inadequate response to the increased thyroid hormone demand. Recent evidence has highlighted the clinical significance of thyroid cysts in terms of their endothelial-related activity. Consequently, it is plausible that thyroid cysts influence the relationship between SCH and height loss.

**Method:**

A prospective-study was conducted in 1,601 Japanese individuals aged 40–74 years with normal serum free triiodothyronine (T3) and free T4 levels. Height loss was defined as being in the highest quartile of annual height decrease.

**Results:**

SCH was significantly and positively associated with height loss among individuals with thyroid cysts, but not among those without thyroid cysts; the sex- and age-adjusted odds ratios (ORs) and 95% confidence intervals (CIs) for height loss were 1.00 (0.56, 1.94) in individuals without thyroid cysts and 2.45 (1.03, 5.81) in those with thyroid cysts. After further adjustment for free T4, atherosclerosis, hypertension, and chronic kidney disease, these associations essentially remained, with adjusted ORs (95% CIs) of 1.00 (0.53, 1.83) and 2.75 (1.12, 6.74), respectively.

**Conclusion:**

Our findings clarify the biological basis of height loss as a cardiovascular risk factor. Endothelial dysfunction is associated with SCH and height loss. Given that urinary iodine concentration has previously been reported to be positively associated with the presence of thyroid cysts and that excess iodine intake is a major cause of SCH in the Japanese population, the observed relationship may reflect an inadequate thyroidal response to increased hormone demand following endothelial injury.

## Introduction

In recent years, numerous past studies have identified height loss as a risk factor for cardiovascular disease [1–5]; however, the underlying biological mechanisms have not been fully elucidated. Since the endothelial condition is the most important determinant of cardiovascular risk, endothelial-related factors might influence height loss.

A previous study of individuals with normal thyroid function (within normal range of serum free triiodothyronine [T3] and free thyroxine [T4]) reported a significant positive association between subclinical hypothyroidism (SCH) and height loss among individuals with low-normal free T4 levels, as well as a non-significant inverse association between SCH and height loss among those with high-normal free T4 levels [6]. However, the mechanisms underlying these seemingly paradoxical associations remain unclear.

Height loss has been reported to be positively associated with endothelial dysfunction, as indicated by structural atherosclerosis [7], and has an inverse association with endothelial repair activity, as measured by circulating CD34-positive cells [8]. These findings suggest that the endothelial status plays a crucial role in the progression of height loss.

Since endothelial progenitor cells (CD34-positive cells) contribute to endothelial repair [9, 10], the number of circulating CD34-positive cells may indicate the capacity to maintain vascular health [11]. Because individuals with SCH have been reported to have lower endothelial progenitor cell counts than those without SCH, and thyroid hormone replacement therapy increases endothelial progenitor cell numbers [12], individuals with SCH and normal-low levels of free T4 have a higher risk of insufficient endothelial repair.

Recent evidence has highlighted the clinical significance of thyroid cysts in thyroid hormone activity and endothelial-related processes such as hypertension and atherosclerosis [13–16]. Excess iodine intake, a known cause of SCH through the inhibition of hormone synthesis via the Wolff–Chaikoff effect [17], may also increase the prevalence of thyroid cysts among older Japanese individuals, given the significant positive association between urinary iodine concentration and both the number and size of thyroid cysts [18].

Since thyroid hormones facilitate endothelial repair [19], an impaired endothelial status associated with height loss [7, 8] may increase the physiological demand for this hormone. Height loss correlates with thyroid function in euthyroid individuals [6], and iodine intake is positively associated with thyroid cysts [18]. Excessive iodine intake causes SCH [17]. Evaluating the relationship between SCH and height loss based on thyroid cyst status could be an effective approach to clarify the impact of the increased physiological demand for thyroid hormones in SCH.

To clarify thyroid cyst-specific associations between SCH and height loss, a prospective study was conducted in individuals with normal thyroid function (serum free T3 and free T4 concentrations within the normal range).

## Materials and methods

### Study population

The methods related to risk surveys, including thyroid examinations, used in this study have been described elsewhere [6, 20, 21].

This study was approved by the ethics committee of Nagasaki University Graduate School of Biomedical Sciences (project registration number 14051404). Informed consent was obtained from all participants. Written consent forms were used to ensure that participants understood the objectives of the study when obtaining informed consent. All procedures involving human participants were conducted in accordance with the ethical standards of the Institutional Research Committee and the 1964 Declaration of Helsinki and its later amendments for comparable ethical standards.

This prospective study included 1,883 Japanese individuals aged 40–74 years from the town of Saza in western Japan. Participants underwent annual medical checkups in 2014, as recommended by the Japanese government.

To minimize the influence of thyroid disease, individuals were excluded if they had a history of thyroid disease (n = 60); had missing data on thyroid function, including thyroid-stimulating hormone (TSH), free T3, or free T4 levels (n = 17); or had T3 (2.1–4.1 pg/mL) or T4 (1.0–1.7 ng/dL) levels outside of the normal range (n = 77).

Individuals without data on atherosclerotic status (n = 1) were excluded. To calculate annual height decrease, at least two height measurements (baseline and endpoint) during the observation period were required. The baseline of the present study was set in 2014; therefore, the participants without height measurements between 2015 and 2022 (endpoint period) were excluded from the analysis (n = 127).

The analysis included 1,601 participants with a mean age of 60.8 years (standard deviation [SD]: 8.9; range: 40–74 years). The mean follow-up was 5.2 years (SD, 2.3 years; interquartile range, 3.0–7.1 years).

### Data collection and laboratory measurements (at baseline)

Trained interviewers collected information on clinical characteristics. Participants were considered to have height loss if they were in the highest quartile of annual height decrease, as described in our previous studies [8, 22, 23]. Blood pressure (systolic and diastolic) was measured in the sitting position using a blood pressure measuring device (HEM-907; Omron, Kyoto, Japan) after at least 5 minutes of rest. High blood pressure was defined as systolic blood pressure ≥140 mmHg, diastolic blood pressure ≥90 mmHg, and/or the use of antihypertensive medication.

Fasting blood samples were collected from all of the participants, and the serum levels of TSH, free T3, and free T4 were measured using chemiluminescent immunoassays (Siemens Healthcare Diagnostics) conducted by LSI Medience Corporation (Tokyo, Japan). The normal ranges for free T3 (2.1–4.1 pg/mL), free T4 (1.0–1.7 ng/dL), and TSH (0.39–4.01 µIU/mL) based on this method have been described elsewhere [24]. Individuals with a serum TSH concentration >4.01 µIU/mL were defined as having SCH. Serum creatinine levels were measured using standard laboratory procedures at SRL Inc. (Tokyo, Japan). Glomerular filtration rate (GFR) was estimated using an established method recently proposed by the working group of the Japanese Chronic Kidney Disease Initiative [25]. CKD was defined as GFR <60 mL/min/1.73 m^2^.

Experienced vascular technicians measured carotid intima-media thickness (CIMT) using a LOGIQ Book XP device with a 10-MHz transducer (GE Healthcare, Milwaukee, WI, USA). The maximum CIMT values of the left and right common carotid arteries were calculated using semi-automated digital edge-detection software (Intimascope; Media Cross, Tokyo, Japan), as described previously [26, 27]. The maximum right and left CIMT values, which did not include plaque measurements, were also calculated. These maximum CIMT values were used in the analysis. Atherosclerosis was defined as CIMT ≥ 1.1 mm [7, 11]. In this study, a thyroid cyst was defined as a structure with a maximum diameter ≥2.0 mm containing no solid components, as in previous studies [16, 20].

### Data collection and laboratory measurements (endpoints)

To evaluate height loss, the following calculation was used: Annual height decrease (Δcm/year) = (Baseline height [cm] − Endpoint height [cm])/(Endpoint year − Baseline year). Height loss was defined as being in the sex-specific highest quartile of annual height decrease.

### Statistical analysis

The status of thyroid cyst-specific clinical characteristics of the study population by SCH is presented as mean ± 1 standard deviation for continuous variables, except for TSH. Because TSH values had a skewed distribution, a logarithmic transformation was performed, and the median [interquartile range] is presented. The prevalence of male sex, hypertension, atherosclerosis, and CKD is expressed as percentages.

Logistic regression models were used to calculate odds ratios (ORs) and 95% confidence intervals (CIs) to determine the association between SCH and height loss.

Three adjustment models were used in this study.

The first model (Model 1) was adjusted for sex and age. Free T4 levels can indicate the status of SCH [28]. According to our hypothesis, rather than the actual levels of thyroid hormone activity, the status of thyroid cysts determined the present associations. The second model (Model 2) was further adjusted for free T4 levels. Since the condition of the vascular endothelium is a critical determinant of the relationship between thyroid function, height loss, and thyroid cysts, direct endothelial status-related factors served as key variables in this analysis. Consequently, while the body mass index, smoking status, drinking status, dyslipidemia, and diabetes are well-known to influence the endothelium, they were not considered confounders in the present study. Given that the presence of atherosclerosis and hypertension determines the phase of vascular remodeling [11, 29], and endothelial repair activity modulates the association between atherosclerosis and CKD [30], we identified hypertension, atherosclerosis, and CKD with age and sexs candidates for adjustment, provided that these factors were closely associated with thyroid function, height loss, and thyroid cysts.

Endothelial repair activity, evaluated by the platelet count and circulating CD34-positive cells [31] determines the association between hypertension and atherosclerosis [32]. Hypertension and atherosclerosis are independent risk factors of height loss [7, 33]. Atherosclerotic status determines the association between thyroid cysts and hypertension [16]. Thyroid cyst status influences the association between TSH and hypertension [13].

Endothelial activity, which is evaluated based on the number of circulating CD34-positive cells determines the association between CKD and CIMT [30]. Thyroid cysts may modify the association between TSH levels and renal function, as evaluated by proteinuria [34]. Therefore, hypertension, atherosclerosis, and CKD may confound the thyroid-specific associations between SCH and height loss. In the third adjusted model (Model 3), adjustments were made for sex, age, free T4, hypertension, atherosclerosis, and CKD.

For additional analyses, we evaluated the influence of anti-thyroid peroxidase antibody (TPO-Ab) titers on the present results by performing the main analysis among individuals with available TPO-Ab data using a model adjusted for sex, age, and TPO-Ab titers. Furthermore, in order to assess the effect of age on the results, we conducted age-stratified analyses using sex- and age-adjusted models to examine the association between SCH and height loss.

To validate the study population in the present study, the goodness of fit was evaluated using the Hosmer–Lemeshow test. Consistent with previous studies [23, 30, 35], p-values <0.05 for the main effects and <0.2 for the interaction terms were regarded as statistically significant.

## Results

Among the study population, 540 participants had thyroid cysts, and 88 had SCH.

### Characteristics of the study population

Thyroid cyst-specific characteristics of the study population according to SCH status are shown in Table 1. Among individuals with and without thyroid cysts, those with SCH had lower free T4 levels than those without SCH. Among individuals without thyroid cysts, the prevalence of hypertension and CKD was higher in individuals with SCH than in those without SCH.

**Table 1:** Characteristics of the study population

	**Thyroid cyst**					

	**(−)**			**(+)**		

	**Subclinical hypothyroidism (SCH)**		**p**	**Subclinical hypothyroidism (SCH)**		**p**
	
	**(−)**	**(+)**		**(−)**	**(+)**	
No. of participants	1000	61		513	27	
Men, %	40.6	42.6	0.240	28.4	33.3	0.586
Age, year	60.0 ± 9.2	62.1 ± 9.0	0.059	62.1 ± 8.1	63.2 ± 8.7	0.459
TSH, (0.39–4.01) µIU/mL	1.51 [1.08, 2.14]*^1^	5.24 [4.50, 6.21]*^1^	<0.001*^2^	1.53 [1.06, 2.17]*^1^	5.35 [4.60, 6.11]*^1^	<0.001*^2^
free T3, (2.1–4.1) pg/mL	3.2 ± 0.3	3.1 ± 0.3	0.111	3.2 ± 0.3	3.1 ± 0.4	0.376
free T4, (1.0–1.7) ng/dL	1.26 ± 0.16	1.18 ± 0.16	<0.001	1.24 ± 0.15	1.12 ± 0.15	0.026
Hypertension, %	36.6	59.0	<0.001	44.1	37.0	0.475
Atherosclerosis, %	8.2	13.1	0.181	11.5	22.2	0.096
CKD, %	16.0	32.3	<0.001	18.5	25.9	0.339

TSH: thyroid-stimulating hormone, T3: triiodothyronine, T4: thyroxine, CKD: chronic kidney disease. Values are mean ± standard deviation. *1: Values are median [the first quartile, third quartile]. *2: Logarithmic transformation was used. Normal range of measurements is ( ). Median values of TSH were 1.3 ng/mL for men and 1.2 ng/mL for women.

### Association between SCH and height loss

The association between SCH and height loss in the entire study population is shown in Table 2.

**Table 2:** Association between SCH and height loss

	**Subclinical hypothyroidism (SCH)**		**p**

	**(−)**	**(+)**	
No. of participants	1513	88	
No. of cases (%)	403 (26.6)	26 (29.5)	
Model 1	Ref	1.37 (0.83, 2.27)	0.225
Model 2	Ref	1.38 (0.83, 2.29)	0.218
Model 3	Ref	1.40 (0.84, 2.33)	0.199

Ref: reference. Model 1: Adjusted only for sex and age. Model 2: Adjusted for sex, age, and free thyroxine (T4). Model 3: Factors adjusted for Model 2 plus atherosclerosis, hypertension, and chronic kidney disease

Although statistical significance was not reached, positive associations between SCH and height loss were observed.

Among the study population, TPO-Ab data were available for 1,329 participants (1,256 without SCH and 73 with SCH). Additional analyses were performed using an adjusted model that included sex, age, and TPO-Ab titer. In this model, the strength of the association is attenuated. The adjusted model showed a similar association, with an odds ratio (95% CI) of 1.08 (0.60–1.95).

### Association between SCH and height loss according to the status of thyroid cysts

Significant positive associations between SCH and height loss were observed only among individuals with thyroid cysts (Table 3).

**Table 3:** Association between SCH and height loss by thyroid cysts

	**Thyroid cyst**					

	**(−)**			**(+)**		

	**Subclinical hypothyroidism (SCH)**		**P**	**Subclinical hypothyroidism (SCH)**		**p**
	
	**(−)**	**(+)**		**(−)**	**(+)**	
No. of participants	1000	61		513	27	
No. of cases (%)	287 (28.7)	16 (26.2)		116 (22.6)	10 (37.0)	
Model 1	Ref	1.04 (0.56, 1.94)	0.907	Ref	2.45 (1.03, 5.81)	0.042
Model 2	Ref	1.05 (0.56, 1.96)	0.887	Ref	2.44 (1.03, 5.83)	0.044
Model 3	Ref	1.00 (0.53, 1.83)	1.000	Ref	2.75 (1.12, 6.74)	0.028

Ref: reference. Model 1: Adjusted only for sex and age. Model 2: Adjusted for sex, age, and free thyroxine (T4). Model 3: Factors adjusted for Model 2 plus atherosclerosis, hypertension, and chronic kidney disease

Among individuals with and without thyroid cysts, 883 (834 without SCH and 49 with SCH) and 446 (422 without SCH and 24 with SCH) had available TPO-Ab data, respectively. Additional analysis was conducted using an adjusted model that included sex, age, and TPO-Ab titers. Because the sample size for this analysis was small, the statistical power was insufficient in order to reach statistical significance. The associations remained essentially unchanged, with adjusted odds ratios (95% CI) of 0.69 (0.32–1.48) for those with thyroid cysts and 2.34 (0.90–6.02) for those without.

Thyroid cyst status showed a notable interaction effect on the association between SCH and height loss. The adjusted p-values were 0.117 in Model 1, 0.116 in Model 2, and 0.059 in Model 3.

### Association between SCH and height loss stratified by age groups

Additional analyses were conducted to evaluate the age-stratified associations between SCH and height loss. In the analyses of all of the participants, a significant positive association between SCH and height loss was observed among individuals aged 60 years and older, whereas a non-significant inverse trend was seen among those younger than 60. The adjusted ORs (95% CIs) for height loss were 2.04 (1.02, 4.08) for individuals aged ≥60 (n = 966) and 0.87 (0.39, 1.98) for those aged <60 (n = 635). When these analyses were further stratified according to thyroid cyst status, similar patterns were observed in both of the age groups. The adjusted ORs (95% CIs) for height loss associated with SCH among individuals without and with thyroid cysts were 1.61 (0.64, 4.02) and 3.17 (1.08, 9.35), respectively, for those aged ≥60, and 0.70 (0.27, 1.86) and 2.05 (0.41, 10.30), respectively, for those aged <60.

### Sensitivity analysis

Using height loss defined as the highest quintile of annual height decrease, thyroid cysts status-specific associations between SCH and height loss were performed for the sensitivity analysis. These results were essentially the same as the main results. Among individuals without thyroid cysts, the adjusted ORs (95% CIs) were 1.04 (0.53, 2.06) for Model 1, 1.02 (0.51, 2.02) for Model 2, and 0.97 (0.48, 1.93) for Model 3. Among individuals with thyroid cysts, the corresponding values were 2.61 (1.05, 6.48), 2.61 (1.04, 6.53), and 2.84 (0.48, 7.25).

## Discussion

The major finding of the present study of Japanese individuals whose serum thyroid hormone concentrations were within the normal range is that SCH was significantly positively associated with height loss among those with thyroid cysts but not among those without thyroid cysts.

Previously, we reported a positive association between SCH and height loss, defined as the highest quintile of annual height decrease, among individuals with normal-low levels of free T4, but not among those with normal-high levels of free T4 [6]. In the present study, we found evidence of a positive association among individuals with thyroid cysts.

A previous prospective study of 363 Japanese men aged 60–69 years found significant inverse associations between the number of circulating endothelial progenitor cells and height loss, defined as the highest quartile of annual height decrease [8]. Therefore, insufficient SCH-related endothelial repair may lead to height loss in the general adult population.

Because endothelial repair is activated when the endothelium is injured, and thyroid hormones contribute to endothelial repair [12], the injured endothelium should increase the demand for thyroid hormones, thereby elevating serum TSH concentration. The presence of SCH indicates a need for thyroid hormone-related endothelial repair.

When the demand for thyroid hormones is elevated, T4 consumption increases because T3 is the active hormone, whereas T4 is inactive, and deiodination, which converts T4 into T3, is stimulated. Among individuals with a sufficient stock of T4, SCH could indicate sufficient endothelial repair activity, whereas among those with a shortage of T4, SCH could indicate insufficient endothelial repair [6].

However, latent thyroid damage may obscure the association between SCH and the level of thyroid hormone demand related to endothelial injury. As latent thyroid damage is accompanied by normal thyroid function, it eludes detection during standard clinical screening.

However, when population-level data were used to categorize participants according to their biological thyroid sensitivity, the presence of this damage was highlighted through clearly discernible patterns. Positive TPO-Ab status, a recognized cause of autoimmune thyroiditis [36], has been reported to be positively associated with TSH levels in euthyroid individuals (those within the normal range for free T3, free T4, and TSH) [21]. Individuals with latent thyroid gland damage require higher TSH levels than those without to maintain normal thyroid hormone levels. Therefore, euthyroid individuals who are TPO-Ab positive are considered to have latent thyroid damage. Furthermore, TPO-Ab titers are inversely associated with the presence of thyroid cysts in individuals with normal thyroid function [20]. These findings suggest that the absence of thyroid cysts in euthyroid individuals may indicate latent thyroid gland damage. Indeed, our previous studies revealed that the presence of thyroid cysts supports thyroid hormone activity [15, 16, 34, 37].

Consequently, the absence of thyroid cysts may be an indicator of latent thyroid damage in euthyroid populations. In our additional analysis using models adjusted for sex, age, and TPO-Ab titer, the magnitude of the association between SCH and height loss was small in the overall population but not in the analyses stratified by the presence or absence of thyroid cysts. Furthermore, although a significant positive association between SCH and height loss was observed among the individuals aged 60 years and older, a non-significant inverse association was found among those younger than 60. After stratifying the analysis by thyroid cyst status, the same thyroid cyst-specific patterns were observed in both groups. These findings partly suggest that stratified analyses based on thyroid cyst status may help reveal underlying thyroid damage.

Therefore, the analysis of individuals without thyroid cysts highlights the influence of latent thyroid damage, which may weaken the impact of endothelial repair activity. Accordingly, in the present study, no significant association was observed between SCH and height loss among individuals without thyroid cysts.

Previous studies have indicated that thyroid cyst fluid contains high concentrations of thyroglobulin, a precursor of thyroid hormones [38, 39]. This suggests that individuals with thyroid cysts may possess an enhanced capacity to meet increased hormonal demands as these cysts serve as functional reservoirs for thyroglobulin. Thyroid hormones play a crucial role in physical growth [40]. A previous study in children and adolescents reported that the ages at which thyroid cysts are most frequently detected are 11 years in males and 13 years in females [41], whereas the mean ages at peak height velocity are 12 years in males and 10 years in females [42]. These observations suggest that the prevalence of thyroid cysts may increase during periods when thyroid hormone demand is elevated. Taken together, these findings raise the possibility that individuals with thyroid cysts may have an enhanced capacity to meet increased hormonal demand, with cysts functioning as reservoirs for thyroglobulin. Ageing is associated with an increased requirement for endothelial repair, and thyroid hormones are known to contribute to endothelial repair processes [11, 12]. Given that individuals with thyroid cysts are significantly older than those without thyroid cysts [13], increased thyroid hormone requirements associated with ageing may contribute to the formation of thyroid cysts.

The positive association between the urinary iodine concentration and thyroid cysts [18] further implies that excessive iodine intake contributes to thyroglobulin accumulation within these cysts. Consequently, SCH in such individuals may be driven by an increased hormonal demand rather than a diminished synthetic capacity. Under the Wolff-Chaikoff effect triggered by excess iodine, thyroid hormone synthesis can be inhibited even in the absence of latent glandular damage, particularly in the presence of thyroid cysts. Under the influence of the Wolff–Chaikoff effect, individuals with thyroid cysts may have an increased ability to boost hormone production in response to elevated hormonal demand, because their hormone supply is suppressed.

**Fig. 1:**
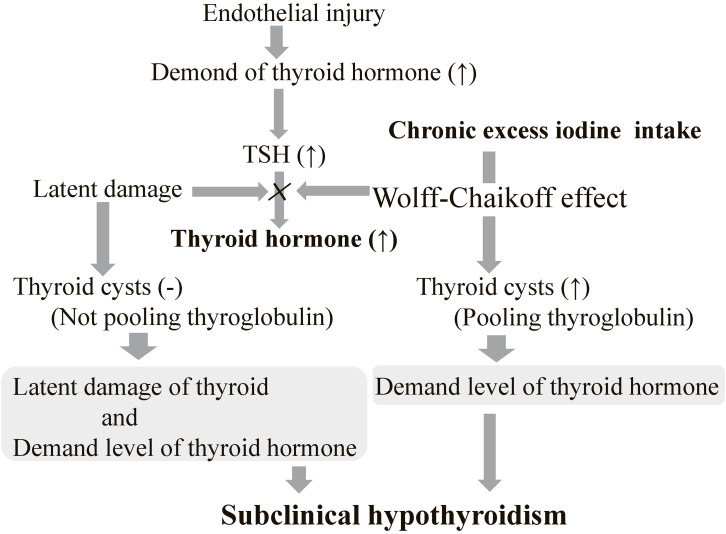
Summary of the potential mechanisms underlying the present results. TSH: Thyroid-stimulating hormone

The potential mechanisms underlying these results are shown in Fig. 1. Excessive iodine intake induces the Wolff-Chaikoff effect, which is linked to thyroid cyst formation. Elevated TSH levels indicate a heightened demand for thyroid hormones. Notably, although latent thyroid damage occurs without cyst formation, both this damage and the increased hormonal demand drive the observed increase in TSH levels.

Our findings have several clinical implications. Although height loss has been identified as an independent risk factor for cardiovascular disease [1–5], the biological mechanisms underlying this association remain unclear. The present findings may serve as a useful tool to clarify the extent to which height loss contributes to the risk of cardiovascular disease. The present findings demonstrate that increased demand for thyroid hormones may cause SCH. Although many studies have reported that treatment for SCH may not be beneficial for older patients [43], no study has distinguished between the increased demand for thyroid hormone-related SCH and latent damage to the thyroid gland-related SCH. Although simple thyroid cysts are generally regarded as clinically insignificant, the present findings suggest that their absence may indicate latent thyroid damage, consistent with our previous studies [13, 14, 16, 20, 34, 37]. Furthermore, because height loss could be a predictor of increased mortality [5], clarifying the mechanism underlying height loss, which the present findings partly support, could be informative for developing strategies to reduce the risk of death.

The limitations of this study warrant further consideration. An efficient cutoff point for defining height loss has not yet been established. Therefore, as in our previous studies [8, 22], height loss was defined in the main analysis as the highest quartile of height decrease per year and in the sensitivity analysis as the highest quintile of annual height decrease. The associations observed in the sensitivity analysis were generally consistent with those observed in the main analyses. As in our previous studies [13, 14, 16, 20, 34, 37], the presence of thyroid cysts was evaluated solely based on their presence or absence. However, the number of thyroid cysts and the size of a given cyst could be important factors affecting the influence of thyroid cysts on thyroid hormone activity. Further investigations incorporating these factors are warranted. Because the urinary iodine concentration may serve as a mediator between SCH and height loss in individuals with thyroid cysts [6, 18], the lack of such data in the present study necessitates further investigation. Future studies should therefore integrate urinary iodine levels to validate our findings and explore the causal pathways involved.

## Conclusion

SCH was significantly associated with height loss in individuals with thyroid cysts, but not in those without. These findings offer a valuable approach for clarifying the biological mechanisms that link height loss to cardiovascular disease risk. Although further research is required, these data indicate that the pathogenesis of SCH may involve two different etiologies: a compensatory response to increased hormone demand, and underlying latent damage to the thyroid gland.

## Abbreviations

SCH: Subclinical hypothyroidism
T4: thyroxine
T3: triiodothyronine
ORs: odds ratios
CIs: confidence intervals
TSH: thyroid stimulating hormone
FT3/FT4: the ratio of free T3 to free T4
CKD: chronic kidney disease
TPO-Ab: anti-thyroid peroxidase antibody
SD: standard deviation
HDLc: high-density lipoprotein cholesterol
CIMT: carotid intima-media thickness

## Declarations

### Availability of data and material

For the studies described in this review, we cannot publicly provide individual data because of participant confidentiality considerations according to the ethical guidelines in Japan. Additionally, obtaining informed consent does not include a provision for publicly sharing the data. Qualified researchers have access to minimal datasets. Please contact the Office of Data Management at ritouken@vc.fctv-net.jp. Information on the data request is available at http://www.med.nagasaki-u.ac.jp/cm/.
